# Risk factors for failed closed reduction in dislocated developmental dysplastic hips

**DOI:** 10.1007/s00264-020-04655-1

**Published:** 2020-06-01

**Authors:** Sebastian Gottfried Walter, Christoph Hans-Jürgen Endler, Anna Christina Remig, Julian A. Luetkens, Rahel Bornemann, Richard Placzek

**Affiliations:** 1 Department of Orthopaedic Surgery, University Hospital Bonn, Bonn, Germany; 2 Department of Cardiothoracic Surgery, University Hospital Cologne, Kerpener Str. 63, 50937 Cologne, Germany; 3 Department of Radiology, University Hospital Bonn, Bonn, Germany

**Keywords:** DDH, Closed reduction, Acetabulum, Congenital, Hip dislocation, Femoral head

## Abstract

**Aim of the study:**

The present study aimed to identify risk factors for unsuccessful CR.

**Introduction:**

Closed reduction (CR) represents the gold standard for treatment of developmental dysplasia of the hip (DDH), but to a minor percentage, it fails to reduce dysplastic hips successfully.

**Methods:**

Seventy-three dysplastic hips underwent closed reduction and post-interventional MRI of the pelvis. MRIs were evaluated for successful reduction of the hip, volumes of femoral heads, and acetabular diameter. Initial treatment results were correlated to AC angles at two years of follow-up. Contralateral, healthy hips served as control.

**Results:**

Out of 73 instable, dysplastic hips, there were nine cases of CR failure. These cases showed significantly increased femoral head volumes (*p* = 0.002) and a significantly (*p* = 0.02) larger ratio of femoral head volume to acetabular opening area. There was no significant difference (*p* = 0.15) in acetabular diameter between both groups. At two years of follow-up, AC angles were significantly (*p* = 0.003) larger and pathologic in cases of CR failure.

**Conclusion:**

Exclusive enlargement of the femoral head is a risk factor for unsuccessful reduction and its ratio to the acetabular opening surface is predictive for CR success in DDH.

## Introduction

Developmental dysplasia of the hip (DDH) is a frequent congenital musculoskeletal deformity with an incidence of 2–4% and hip luxation as its severe form in 0.4–0.7% of all cases [1, 2]. The current gold standard for the early treatment of hip dislocation (in patients younger than 12 months) is fluoroscopic-guided closed reduction (CR) with subsequent spica cast (Fettweis cast) immobilization [3]. Although this technique is associated with a high success rate, there is dissent on predisposing risk factors for failed CR. In the past, hypertrophic ligaments, an inverted labrum, or adipose tissue have been claimed to obstacle reposition, which could not be verified in recent studies [4, 5].

The present study was set up to evaluate infantile dysplastic hips for risk factors resulting in treatment failure and to correlate post-interventional MRI data to two year radiological follow-up data according to the Tönnis classification.

## Methods

This is a retrospective study including 65 consecutive patients (73 DDH hips) with hip dislocation due to DDH. All instable or dislocated hips underwent closed reduction (CR) treatment as first-line treatment in the time between 2013 and 2018.

The study has been performed in accordance with the ethical standards laid down in the 1964 Declaration of Helsinki and was approved by the local ethics committee.

### Patients

During the recruitment phase, 28,600 screenings for detection of DDH were performed at our institution as part of a general nationwide screening program either at U2 examination (nationwide screening program for infants; 3rd–10th day after birth in case of risk factors for DDH [6]) or U3 examination (3rd–8th week after birth). Inclusion criteria were positive clinical examination (positive Galeazzi and Ludloff sign as well as abduction inhibition) and sonographic confirmation according to the Graf criteria for hip subluxation [7].

Exclusion criteria were underlying diseases or syndromes associated with teratologic or secondary neuromuscular hip dislocation.

Patients underwent CR the day or the day after the diagnosis had been made.

### Treatment

Closed hip reduction was performed in general anaesthesia. Patients were placed in supine position, and isotonic sodium-chloride followed by contrast medium was injected into the dislocated hip joint through a posterior approach as described elsewhere [8]. Hips were then repositioned under fluoroscopic guidance, and subsequently, a hip spica cast was applied with legs in at least 90° flexion and 60° abduction according to the position of maximum stability. Subsequently, an MRI of the pelvis was performed to control for joint containment. If the hip joint was dislocated, the cast was opened immediately and closed reduction was redone or open joint reposition was performed. If the MRI showed successful hip reposition, the cast was left for four to six weeks. If clinical and sonographic examination showed a successfully repositioned and stable hip after cast removal, a Tuebinger flexion abduction splint was prescribed and worn until hips were matured (Graf type 1). In case of continued unstable hips, a further closed reduction was done [8]. Patients were followed up at two years of age (and scheduled for 5- and 1-year follow-up).

### MRI evaluation

**Fig. 1:**
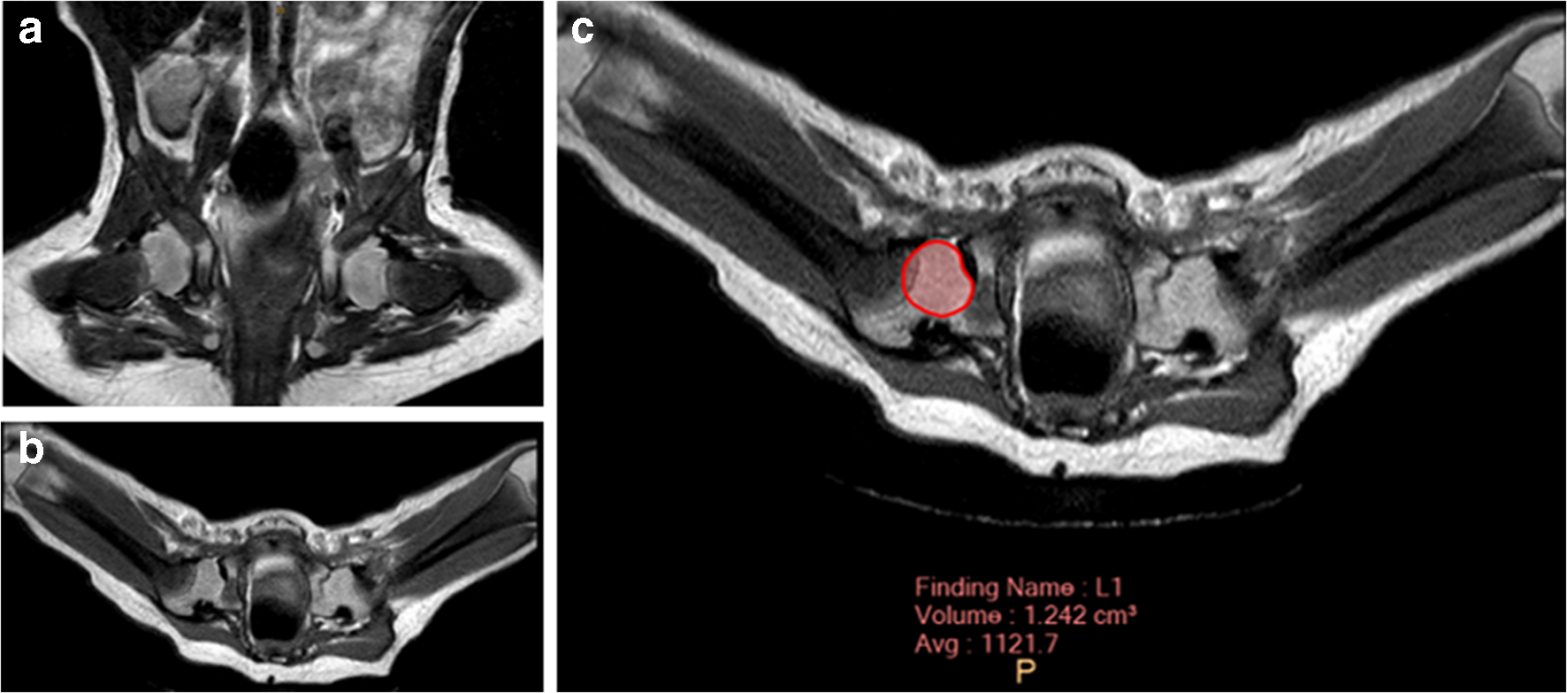
**a** A coronal projection of a DDH of the right side and **b** its corresponding transversal projection after successful closed reduction. **c** The principle of measurement for femoral head’s volume at one level

**Fig. 2:**
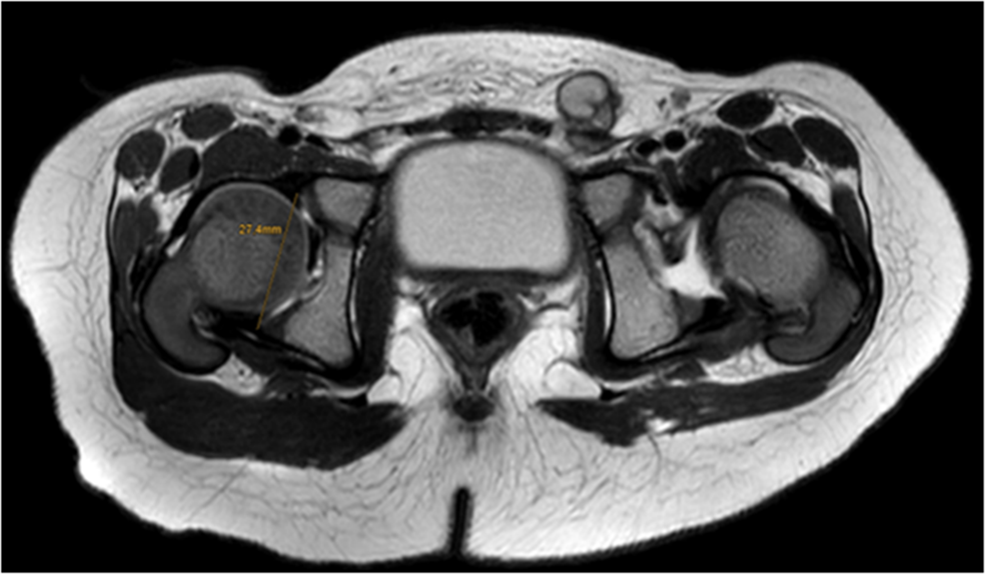
Acetabular diameter of both sides was measured in the transversal plane of the largest diameter. The hip of the left side was dislocated and CR was successful

For MRI, patients were placed in supine position with the previously applied pelvic cast. In most cases, images were obtained without additional anaesthesia using a Philips Ingenia System (Philips Healthcare, Best, The Netherlands) at field strengths of either 1.5 Tesla (T) or 3.0 T. T2-weighted turbo spin echo (TSE) sequences in the transverse and coronal plane were used to evaluate the correct reposition of the hip. The slice thickness was set to 2.5 mm. For measuring femoral head volumes (Fig. 1) and acetabular diameters (Fig. 2), IntelliSpace Portal (Version 10.1, Philips Medical Systems, Best, Netherlands) was used. For volumetry, the femoral head was segmented in each axial slice. The largest acetabular diameter was also measured in axial sequences. Acetabular opening area was calculated by *A*_o_ = πd^2^/4. All measurements were performed by three radiologists with routine in MR imaging for both DDH and contralateral, healthy hips. For estimation of intra-observer reliability, measurement was repeated six months after the initial measurement.

### Evaluation of two year follow-up

At two years of age, patients were followed up clinically (positive Galeazzi and Ludloff signs, abduction inhibition, gait pattern) and radiologically (a.-p. pelvis x-ray). For evaluation of the later, two trained orthopaedic fellows measured AC angles of patients’ hips. For estimation of intra-observer reliability, measurement was repeated by on orthopaedic fellow three months after the initial measurement. For measuring angles and distances, IMPAX FX (Version 3.17, AGFA Healthcare) tools were used.

### Statistical analyses

Data were analyzed using SPSS Statistics 25.0 (SPSS Inc., Chicago, IL). Descriptive statistics, including arithmetic mean value and standard deviation, were calculated. Data are given as means ± standard deviation (SD) and ranges, if not indicated otherwise.

The nonparametric Mann-Whitney *U* test was performed for metric, not normally distributed data. The Students’ *t* test was performed for metric, normally distributed variables. Multivariate analysis was performed for identification of possible risk factors for CR failure. For inter- and intra-observer reliability of AC angles, interclass coefficients (ICCs) were calculated. Inter- and intra-observer reliabilities of the individual categorical features were determined using kappa statistics. Any probability value of less than 0.05 was considered as statistically significant (*p* = 0.05).

## Results

### General results

Sixty-five consecutive patients (52 female, 13 male) were included in this study of which none suffered from an underlying disease or syndrome associated with dysplasia of the hips. There were 43 patients with a hip dislocation at the left and 14 patients with a hip dislocation at the right side. Another eight patients had bilateral dislocated hips. The mean age of patients at the time of CR was 37.4 days (range: 1–191 days).

There were a total of 73 dislocated hips that were treated by closed hip reduction. Analysis was done for 130 hips (65 patients) of which 73 were primarily unstable and 57 primarily stable (contralateral side; Table 1). Nine of 73 primarily unstable hips were analyzed separately as closed reduction failed.

**Table 1:** Overview of key outcome measures of closed reduction MRI assessment

	SH (*n* = 57)		Primarily unstable hips				Significance (*p*) by *t* test	
	Left	Right	SRH (*n* = 64)		FRH (*n* = 9)		SH vs. SRH	SRH vs. FRH
			Left	Right	Left	Right		
Femoral head in mm^3^	2.1 ± 0.7	2.3 ± 0.8	2.3 ± 0.8	2.2 ± 0.7	3.2 ± 1.6	4,5 ± 3.5	0.623	*0.002
Acetabular diameter in mm	1.3 ± 0.6	1.4 ± 0.2	1.4 ± 0.2	1.4 ± 0.3	1.5 ± 0.4	1.6 ± 0.2	0.544	0.150
AC at 2 years	24.8 ± 3.1	24.3 ± 2.9	25.1 ± 4.4	23.4 ± 3.8	31.3 ± 1.9	33.5 ± 1.5	0.547	*0.003

Significant values are marked by “*”. Significances were tested for primarily stable hips (SH; contralateral “healthy” hips) vs. successfully reduced hips (SRH). The later (SRH) were tested for significant differences against failed reduced hips (FRH)

**Fig. 3:**
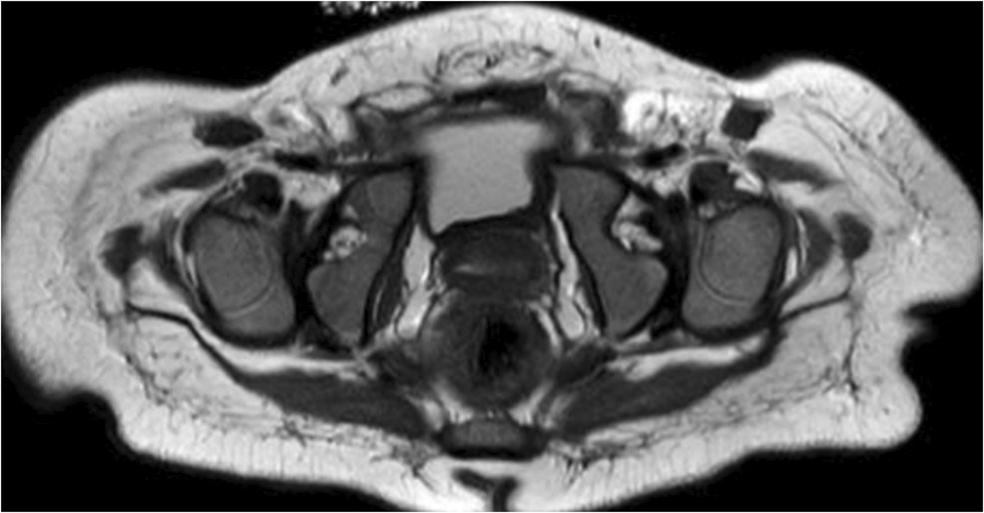
Case with bilateral congenital dislocation of the hip joints. While the right side could be repositioned by an additional CR trial, the left side underwent open reduction, and subsequently acetabular osteotomy

If closed reduction failed, treatment was repeated or eventually converted to open reduction. Primary closed reduction failure was counted as failure even if secondary (subsequent) closed reduction was successful (*n* = 4) (Fig. 3). In seven patients, failed reduction was detected in MRI, while in the other two patients, hips dislocated secondarily after the MRI was performed. Of these nine patients, two had undergone previous closed reduction and one had undergone additional open reduction elsewhere previously (*n* = 1). On average, these nine patients were significantly older than the rest of the cohort (88.6 days (5–191 days); *p* < 0.001). In three of these nine patients, avascular necrosis (AVN) was observed in the course of the treatment [9]. Femoral head size was not significantly larger (*p* = 0.53) in AVN hips than in the other six hips that had failed CR. AVN was not observed in successfully reduced hips.

Another ten patients had undergone previous treatment by usage of a Pavlik harness or a Tuebinger flexion-abduction-splint for less than two weeks as they were treated *ex domo* first and were referred to our centre. None of the patients underwent previous longitudinal or overhead traction.

### Graf classification

Initial unstable hip types according to Graf’s classification were type IIc (*n* = 4), type “D” (*n* = 6), type III (*n* = 24), and type IV (*n* = 39). All initially unstable but successfully reduced hips showed type I (*n* = 64) 4 to 8 months after closed reduction. Initial stable hips showed either type I (*n* = 19), type IIa (*n* = 33), or type II c (*n* = 5) according to Graf’s classification.

### Femoral head volume

There was no significant difference of the femoral head volume between primary stable and unstable dysplastic hips (*p* = 0.623). In cases of CR failure, femoral head volumes were significantly larger (3.7 ± 2.1 mm^3^) than in successfully reduced hip joints (2.3 ± 0.8 mm^3^; *p* = 0.002). ICC values for intra- and inter-observer reliability were 0.83 and 0.80, respectively.

Femoral head volume did not significantly differ between different severities of hip dislocation (according to Graf; Table 2).

**Table 2:** There was no significant differences in femoral head volume between different severity grades according to the Graf classification (primarily stable hips/instable hips)

Severity according to Graf	Femoral head volumes in mm^3^	*p*
I (19/0)	2.2 ± 0.6	
IIa (33/0)	2.2 ± 0.8	0.68
IIc (5/4)	2.3 ± 0.4/2.3 ± 0.6	0.49/0.52
D (0/6)	2.2 ± 0.7	0.65
III (0/24)	2.6 ± 0.7	0.37
IV (0/39)	2.5 ± 0.8	0.43

### Acetabular diameter

There were neither significant differences between primary stable and successfully reduced hips (*p* = 0.544) nor between the latter and failed reduced hips (*p* = 0.150). ICC values for intra- and inter-observer reliability were 0.88 and 0.82, respectively.

### Femoral head volume/acetabular opening area ratio

This ratio showed a significant difference (*p* = 0.02) between successfully reduced hips (1.4 ± 0.09) and failed reduced hips (2.0 ± 0.22). There was no significant difference between primary stable hips (1.4 ± 0.23) and successfully reduced hips (*p* = 0.47).

### AC angle at two years

At two years of age, there was no significant difference regarding the AC angle between primary stable and successfully reduced hips (*p* = 0.547). Those hips were graded as “normal” according to the age-dependent dysplasia scale by Tönnis [10]. AC angles were significantly larger in failed reduced hips (*p* = 0.003) and had mean values of 31.9° ± 1.8° (grade 3 or “severe pathological” according to Tönnis). ICC values for intra- and inter-observer reliability were 0.84 and 0.79, respectively.

### Clinical results at two years

At two year follow-up, Galeazzi sign, Ludloff sign, and abduction inhibition were negative in all cases of successful CR. Gait patterns broadly varied; yet, all patients were able to stand and walk.

### Reduction obstacles

In none of the cases of failed closed reduction, soft tissue obstacles were detected in MRI as reason for failure. However, instable hips showed a deformed and bulged labrum, while primary stable hips did not show cartilaginous deformations.

Multivariate analyses found an increased risk of CR failure in cases of increased femoral head size with respect to the contralateral “healthy” hip (AOR 4.27, *p* = 0.016) and increased femoral head volume/acetabular opening area ratio (AOR 4.81, *p* = 0.002). Patient’s age was not identified as solitary risk factor (AOR 10.24, *p* = 0.27).

## Discussion

It is commonly acknowledged that early diagnosis and treatment of DDH are crucial for subsequent physiologic maturation of the hip joint and avoidance of early joint degeneration. Although treatment success can be monitored by sonography, clinical examination, and subsequently by x-ray, specific morphologic criteria that correlate with treatment failure have not yet been defined.

There are different techniques for treatment of DDH. Traction techniques as well as usage of Pavlik harnesses allow for intermitting sonographic controls according to Graf. These techniques, however, are limited in therapeutic success due to limited initial cartilaginous maturation [11, 12] and evidence for an increased risk of developing avascular necrosis of the femoral head [13–15]. Although the mentioned techniques are known to be associated with these limitations, closed reduction has become gold standard for the treatment of unstable developmental dysplastic hips due to better success rates only recently [16]. So far, existing studies on closed reduction present small sample sizes [4] or do not assess success of reduction immediately after spica casting [17] although MRI is widely accepted for this indication [9]. Furthermore, different inclusion criteria and patient characteristics (age, diagnostic technique, etc.) limit comparability of studies available.

This study presents one of the largest MRI-evaluated cohorts of patients undergoing closed reduction. Treatment success was evaluated short-term by MRI and mid-term by correlating initial treatment success to AC angles at two years of patients’ age.

In this study, an overall success rate of about 90% was detected for closed reduction, which is in correspondence to other studies assessing closed reduction based on different parameters [15, 18, 19].

As reported previously, soft tissue obstacles almost never account for unsuccessful CR in cases of DDH [4, 5]. Therefore, different morphologic correlates were to be identified to explain for treatment failures. In cases of CR failure, a significantly enlarged volume of the femoral head was measured. The corresponding acetabulum and its diameter, however, did not show any disproportionate growth. The resulting mismatch of femoral head volume and acetabular opening area was identified as mechanical obstacle hindering joint reduction. It can be hypothesized that hip dislocation in DDH allows the femoral head to grow disproportionally faster than the corresponding part of the pelvis. Therefore, patient’s age at the time of CR is confounding for success probability as the process of disproportionate growth is assumed to be time-dependent. Although this study presents a large cohort, it is difficult to define a cut-off value for the “femoral head volume/acetabular opening area ratio”. Nevertheless, in the described cohort, CR was unsuccessful in cases with a ratio larger than 1.8.

Considering that AC angles of successfully reduced and primary stable hips were similar at two years of follow-up, it can be claimed that physiologic maturation of the hip joint is no longer impaired once the regular anatomy has been restored. If anatomic restoration was not possible due to a size mismatch between femoral head and acetabulum, hip maturation was impaired, which was indicated by pathological AC angles.

This study has several strengths as it (a) evaluates a large number of CR treated DDH hips, (b) presents novel MRI parameters for the evaluation of DDH hips after reduction, (c) correlates initial treatment result to mid-term radiological outcomes, (d) is representative for paediatric orthopaedic centres in countries where a general screening for DDH is mandatory. Its single centre and retrospective character may limit the study evidence. Pre-interventional and follow-up MRI would be desirable. Yet, we had great ethical concern to perform additional MRIs as the risk of an additional anaesthesia was not justifiable and not according to good clinical practice.

It may be criticized that femoral head volume was not adjusted to age. Our patient cohort, however, was very young and different individual growth and body size percentiles are likely to conceal significant age-femoral head volume correlations and thus adjustment may be misleading or insignificant. In this study, patient’s age seems very young at the time of CR; however, the authors believe early casting to be beneficial as “physio-motoric” development is not impacted and delayed in contrast to casting during the time of “verticalization”. One patient underwent CR at one day of age, as hip instability was diagnosed at the day of birth (Graf type IV) due to presence of risk factors. This may seem aggressive, yet CR was successful without complications, the cast was removed after 4.5 weeks, and the hip was matured at eight weeks of age (Graf type I). According to treatment standards in German speaking countries, treatment starts as early as possible [20, 21].

In order to gain more evidence, current research is ongoing and patients are followed up long-term. Future studies should investigate whether patients at risk for treatment failure would benefit from pre-operative MRI.

In summary, early diagnosis and treatment of congenital hip dislocation are essential for a physiologic and regular maturation of the hip joint as joints that remained in dislocation for several weeks develop a mismatch between femoral head and acetabulum.
