# Correction to: Risk factors for failed closed reduction in dislocated developmental dysplastic hips

**DOI:** 10.1007/s00264-021-05124-z

**Published:** 2021-09-09

**Authors:** Sebastian Gottfried Walter, Christoph Hans-Jürgen Endler, Anna Christina Remig, Julian A. Luetkens, Rahel Bornemann, Richard Placzek

**Affiliations:** 1 Department of Orthopaedic Surgery, University Hospital Bonn, Bonn, Germany; 2 Department of Orthopaedic Surgery and Traumatology , University Hospital Cologne, Kerpener Str. 63, 50937 Cologne, Germany; 3 Department of Radiology, University Hospital Bonn, Bonn, Germany

**Correction to: International Orthopaedics (2020) 44:2343–2348**

**https://doi.org/10.1007/s00264-020-04655-1**

The article **“Risk factors for failed closed reduction in dislocated developmental dysplastic hips”**, written by Sebastian Gottfried Walter, Christoph Hans-Jürgen Endler, Anna Christina Remig, Julian A. Luetkens, Rahel Bornemann and Richard Placzek, was originally published Online First without open access. After publication in volume 44, issue 11, page 2343–2348, the author decided to opt for Open Choice and to make the article an open access publication. Therefore, the copyright of the article has been changed to © The Author(s) 2020 and the article is forthwith distributed under the terms of the Creative Commons Attribution 4.0 International License (http://creativecommons.org/licenses/by/4.0/), which permits use, duplication, adaptation, distribution and reproduction in any medium or format, as long as you give appropriate credit to the original author(s) and the source, provide a link to the Creative Commons license, and indicate if changes were made. The images or other third party material in this article are included in the article’s Creative Commons licence, unless indicated otherwise in a credit line to the material. If material is not included in the article’s Creative Commons licence and your intended use is not permitted by statutory regulation or exceeds the permitted use, you will need to obtain permission directly from the copyright holder. To view a copy of this licence, visit http://creativecommons.org/licenses/by/4.0. Open access funding enabled and organized by Projekt DEAL.

The original article was corrected.

